# Is Fat Deposition of Renal Sinus a Concomitant Finding to Fatty Liver Disease? The First Study Regarding the Relationship Between Kidney and Liver Fat Content with Non-Contrast Computed Tomography

**DOI:** 10.51894/001c.32411

**Published:** 2022-02-24

**Authors:** Emrah Doğan, Ferda Bacaksızlar Sarı

**Affiliations:** 1 Radiology Mugla Sıtkı Kocman University; 2 Mugla Sıtkı Kocman University Education of Research Hospital

**Keywords:** Renal fat deposition, Fatty liver disease, Computed tomography, Hepatic steatosis

## Abstract

**INTRODUCTION:**

It has been established that abnormal fat deposits are associated with fat deposition in other abdominal regions and linked to obesity, diabetes mellitus, hypertension, vascular and metabolic diseases. This study aimed to determine whether there was a relationship between fat deposition of the renal (i.e., kidney) sinus (FRS) and fatty liver disease (FLD) in a sample of adults. The authors hypothesized that FRS could be a diagnostic finding associated with Hepatosteatosis (HS) in a sample of younger patients. This study was the first apparent investigation of this possible phenomenon.

**METHODS:**

A convenience sample of 92 adult patients of which 19 (20.7%) were females and 73 (79.3%) were males, and with a mean age of 30.19 (SD = 6.00) were included. The authors calculated Hounsfield Units (HU) (i.e., relative quantitative measurement of radio density) of patients’ livers and spleens on non-contrast computed tomography (CT). Liver and spleen differences < 10 HU were considered steatosis (FLD). The authors stratified sample patients into two analytic subgroups according to the presence of FLD or not and compared them based on their FRS widths.

**RESULTS:**

In the FLD subgroup (N = 48), the difference of HU values between liver and spleen was -5.19 (SD = 11.32), with a range of -38 - 8 HU, while, in the non-steatosis subgroup (N = 44), the mean difference was 16.36 (SD = 3.90), range of 11 - 26 HU. The average diameter of FRS width was 12.5 mm in those patients with steatosis (FLD subgroup) although 9.3 mm in non-FLD patients. (p = 0.02)

**CONCLUSIONS:**

Based on these results, FRS may be able to be used by radiologists as an ancillary method in the detection of hepatic steatosis in younger adults. The effectiveness of premedical processes (e.g., exercise and diet modification) can also be increased by non-radiologists after detection of lower-grade HS.

## INTRODUCTION

It has been established that abnormal fat deposits in the body are associated with development of obesity, diabetes mellitus, hypertension, vascular and metabolic diseases, and thus, contribute to population morbidity and mortality.^1^ Two abnormal fatty deposit regions of the body have received the greatest attention: hypertrophy of the subcutaneous/visceral fatty tissue and hepatic steatosis (HS), i.e., excessive fat build up in the liver.^1,2^

Although HS is among the most reported findings in abdominal computed tomography (CT) scans, detection rates have been concluded to be well below actual values.^3,4^ According to a Canadian 2016 study conducted by Wells et al., only 40% of all fatty liver disease (FLD) is reported on CT by radiologists.^3^ This same study estimated that 85% of family physicians were unaware of the presence of HS in their patients.^3^ The main reason for this lack of awareness is that in terms of radiologic imaging, the appearance of liver density may appear grossly normal on CT scans.^3^ However, the presence of ancillary findings in surrounding organs may increase the diagnostic rate of FLD by directing the radiologist to measure Hounsfield Units (HU), a quantitative scale used to describe radiodensity, during CT assessments.^3,5^

Studies concerning CT fat deposition patterns that accompany FLD have been quite limited to date. The correlation between fat deposition of renal sinus (FRS), i.e., cavity within the kidney, and FLD has not yet apparently been investigated. In 2020, Yalçın et. al., compared hypertrophy of visceral adipose tissue and adiposity of peripheral organs without mentioning FRS.^5^ In another 2020 article, Yamato, et. al. studied the relationship between hypertrophy of subcutaneous/visceral adipose tissue and FLD.^6^ The renal hilum is among the minor fat deposition points of the abdomen.^7^ The authors of this paper therefore concluded similarly to the 2020 Yalçın, et. al.^5^ study that these fatty deposit relationships could best be evaluated using CT imaging. Further, the presence of a HU difference <10 between the liver and spleen can be used to assess FLD.^3^

### Study Purpose

The purpose of this study was to investigate the relationships between FRS on CT scans and FLD. With a normal aging process, fatty tissue in the renal hilum gradually increases and may mask the findings of FRS due to FLD.^5^ The authors hypothesized that FRS could be a diagnostic finding associated with HS in an available sample of younger patients.

## METHODS

Before data collection, the authors obtained 2021 approval from their university IRB. Sample images were selected among the non-contrast abdominal CTs performed between February 2020 and July 2021.

**Figure 1.**
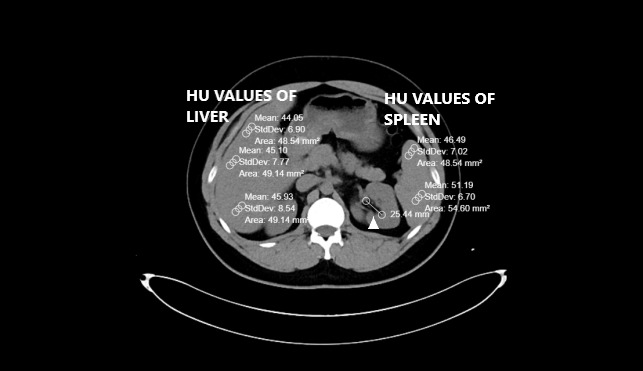
Calculation of HU values ​​of liver and spleen as well as FRS diameter in patient with steatosis (Arrowhead). Pay attention to the difference between the liver and the spleen HU.

**Figure 2.**
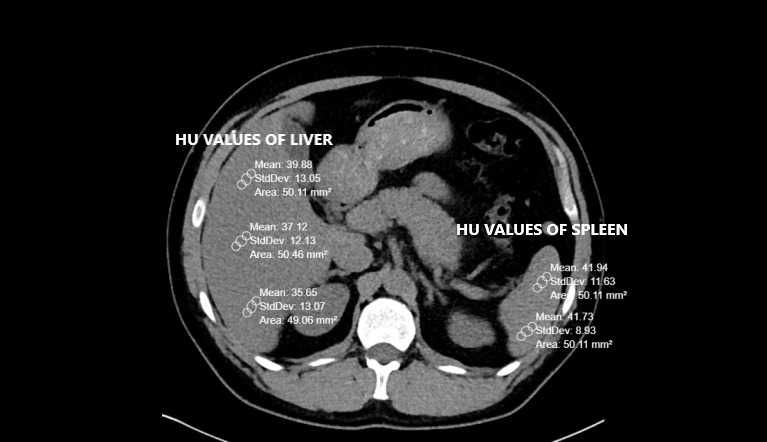
Calculation of the HU values of the liver and spleen as well as the FRS diameter in the patient without steatosis. Pay attention to the difference between the liver and the spleen HU.

Pre-existing CT scans had been performed with a 256-slice multi-detector CT scanner (Somatom, Siemens Healthcare, Erlangen, Germany). Abdominal CT images were reconstructed by the authors on coronal, axial and sagittal planes. All CTs were taken without contrast and in a supine position. The other CT scan parameters were as follows: rotation time, 0.35s; thickness, 1mm; FOV (field of view), 30-40 cm.^8,9^ Liver and spleen HU values were calculated from a minimum of three different points. The presence of a HU difference <10 between the liver and spleen is one of the two FLD criteria used in the study.^3^ Therefore, it was important to obtain spleen values along with the liver. Region of interest (ROI) was selected as approximately 50 units. Liver CT window (width (W):150 Hounsfield units (HU), length (L):30 HU) was used during the measurements (Figures 1 and 2).

The livers and spleens of sample patients were also evaluated in the abdominal window (W:400 HU L:50 HU). The measurement points were chosen from different liver segments. Measurements were not taken from regions where focal fat deposition was observed (e.g., periportal areas and around the gallbladder). Peripheral area measurements were preferred because of hilar vascularity, when measuring spleen HU.

If the difference in CT attenuation between liver and spleen was less than 10 HU, the patient was included in the HS group. Otherwise, they were added to the non-steatosis group.^4^ After determining each sample patient’s subgroup, the FRS width was also calculated in both kidneys. Values between -30 and -70 HU (around -50) have been accepted for fat density.^10^

The largest diameter in the axial plane was considered. A data scale containing the HU values of the liver, spleen, the HU difference of two organs and the largest diameter of FRS (cm) was calculated.^5,11^ Each image was independently assessed and liver and spleen HU units were independently calculated by two experienced radiologists. In case of contradictory results, such images were re-evaluated by both radiologists together.

The analytic sample was stratified into two independent subgroups (i.e., patients with HS and patients without steatosis). A pre-analysis minimal sample size power analysis had been conducted using *G-power 3 software*, (https://www.psychologie.hhu.de/arbeitsgruppen/allgemeine-psychologie-und-arbeitspsychologie/gpower) indicating that a total analytic sample of 72 (i.e., 36 per sample subgroup) would provide the authors with 0.80 1-β power to detect meaningful sample subgroup differences.

Data were stored on a Microsoft Office Excel spreadsheet file (Excel 2010, Microsoft), and a statistical analytic software (i.e., SPSS, version 22.0, IBM) was used to conduct analytic procedures. Continuous variables were expressed as mean ± SD (Standard Deviation) values. Categorical variables were expressed as counts and percentages. The Student’s T-test was conducted to compare means of continuous variables. Pearson chi-square (χ2) procedures were completed to evaluate the relationship between categorical variables. A coefficient Alpha p value of less than 0.05 value was observed as statistically significant.

## RESULTS

A total of 120 patients were first evaluated in terms of HS. The authors excluded a subset of 28 (23.3%) patients from the study sample for the following medical reasons: 12 (10.0%) patients had kidney stones, seven (5.83%) had parenchymal atrophy, two (1.60%) had a history of corticosteroid use. Also, two (1.60%) additional patients were excluded from the study due to known splenic diseases (i.e., comparison of liver and spleen was one of the main parameters of the study and splenic diseases would have affected study results). In addition, one patient (0.80%) CT had imaging motion artefacts that prevented evaluation. Four (3.3%) other excluded patients had mosaic/focal steatosis. This left a total of 92 patients for the analytic sample. A predominance of males 73 (79.3%) in the total sample was noted while 19 (20.7%) were female.

### HS subgroup

This subgroup was comprised of 48 patients consisting of 41 (85.4%) males and 7 (14.6%) females. The mean age of the HS subgroup was 30.06 (SD = 5.36) and ranged from 18 – 39 years of age. Mean liver HU values averaged 41.14 (SD = 12.93) and ranged from 2 – 57. The mean splenic HU value was 45.62 (SD = 4.16) and ranged from 34 - 54. The mean differences between liver and spleen HU values were -5.19 (SD = 11.32) and ranged from -38 - 8.

### Non-HS subgroup

This subgroup was comprised of 44 patients with 32 (72.8%) males and 12 (17.2%) females. The mean age for the Non-HS subgroup was 30.34 (SD = 6.70) and ranged from 18 – 46 years of age. Mean liver HU values averaged 61.23 (SD = 4.30) and ranged from 54 - 69. The mean splenic HU value was 45.07 (SD = 4.13) and ranged from 33 - 52. The mean differences between liver and spleen HU values were 16.36 (SD = 3.90) and ranged from 11 - 26.

The two following main CT evaluation criteria were taken into account and compared.

**Criterion I:** The measured HU value was less than 40.

**Criterion II:** The difference between liver and spleen HU values was less than 10.

Considering all 92 sample patients, HU values of the livers were 50.75 (SD = 14.02) and ranged from 2 - 69. HU values of the spleens were 45.36 (SD = 4.13) and ranged from 33 – 54. However, 13/44 (29.5%) of the patients in the HS group had a liver HU value < 40. There was discrepancy between the two criteria. This comparison is shown in Table 1.

**Table 1.** The number of patients and rates in the HS and non-steatosis groups according to criterion I and II (comparison)

**Criteria I/II**	**Liver value** **(< 40 HU)**	**Liver value** **(> 40 HU)**	**Spleen** **(30 - 60 HU)**
**Steatosis (N = 44)**	29.55% (N = 13)	70.45% (N = 31)	100% (N = 44)
**Non-steatosis (N= 48)**	-	100% (N = 48)	100% (N = 48)

**Figure 3.**
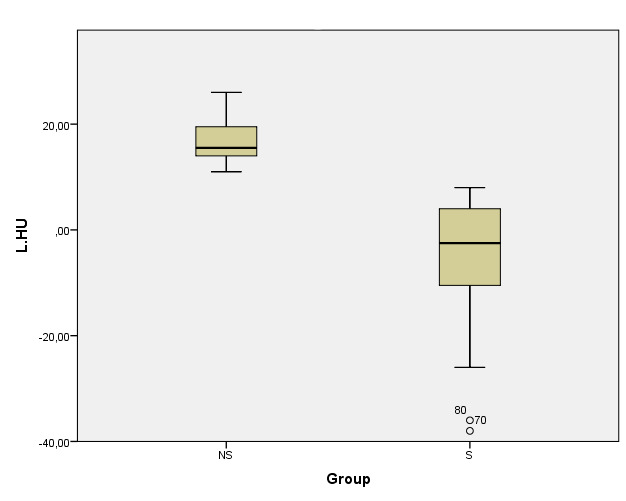
The box plot graphic shows difference between the two groups according to Criterion II

Figure 3 depicts the notable differences between HU values of the liver and the spleen in the HS and non-steatosis sample subgroups.

FRS widths were also assessed in both subgroups. The table below shows means, standard deviations and variances (Table 2).

**Table 2.** Statistical values of FRS width in the HS and Non-HS subgroups.

**Subgroup**	**Mean (cm)**	**SD**	**Variance**
**Steatosis (N = 44)**	12.54	6.00	36.01
**Non-steatosis (N= 48)**	9.35	6.36	40.46

**Figure 4.**
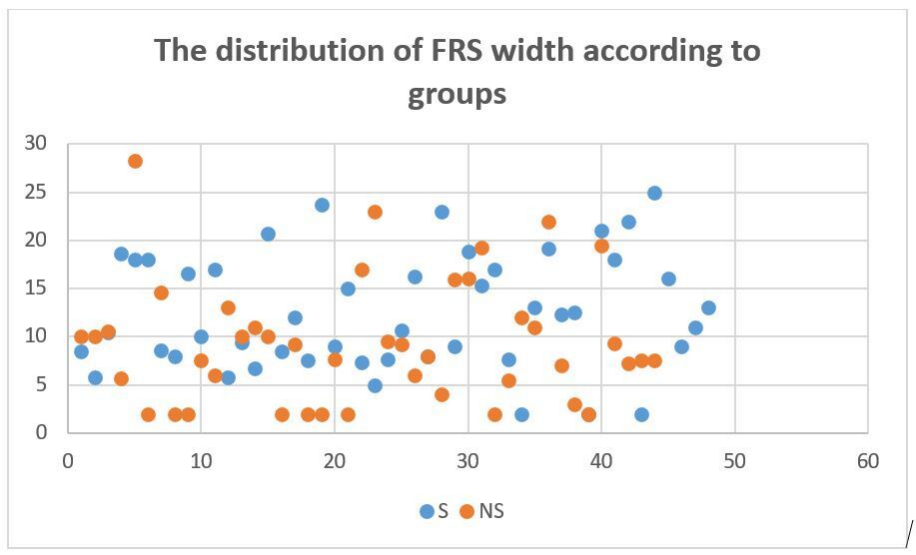
Scatter plot of FRS of HS and non-HS sample subgroups. **Blue** dots = HS patients **Orange** Dots= Non-HS patients

The means were compared with independent student’s T test. The width of the FRS of steatosis groups was significantly higher than non-steatosis group (f: 0.162, ddl: 90, t value: -2.477, the mean of difference: -3.19205 difference error standard: 1.28892, P value: 0.02). A scatter plot distribution of FRS width is shown in Figure 4.

## DISCUSSION

NOTE: The term “fatty infiltration” of the liver has been preferred to defining fat storage of the liver since HS is an intracellular condition (i.e., there is no fatty infiltration of the extracellular matrix-limited to hepatocytes).^12,13^ However, since this phrase is widely used in the medical literature, we have incorporated this condition into HS in this discussion section.

As indicated in earlier studies, diffuse HS is a common condition affecting over 25% of the population.^3–7^ This condition has been shown to affect males more often than females.^14^ The findings of our study were similar as 85.6% of sample patients with HS were males.

This condition is often associated with common diseases and metabolic comorbidities (e.g., obesity, type II Diabetes, Hyperlipidemia, Hypertension and Metabolic Syndrome).^15–17^ Confirming the presence of HS in higher risk patients is therefore often crucial for the monitoring and treatment of fatty liver disorders in conjunction with these comorbidities.^18^

However, there are some potential measurement pitfalls in evaluating patients for HS. Failing to identify mild degrees of HS may be increased in patients with concomitant chronic kidney disease due to the echogenicity (i.e., ability to reflect ultrasound waves) of the kidneys.^19^ Although dynamic MRI imaging may readily confirm moderate to severe HS, MRI can be very weak for detection of lower-grade HS.^20–22^

Contrast-enhanced CT is infrequently used during assessments of HS because the procedure contrast can often veil HS signs, making non-contrast CT superior.^20,21^ Although all imaging methods may have limited sensitivity for the evaluation of early grade steatosis,^20^ overall CT remains more effective than other radiologic methods in the evaluation of low-grade steatosis.^23–25^

Various criteria have been proposed for the diagnosis of HS on CT without contrast to detect HU values < 40 in the parenchyma.^21,25–28^ We would like to suggest that the two criteria we used for this study provided a more general imaging approach concerning liver fat depositions.

Aging-related increases in fatty tissue in the kidneys can also occur when the renal parenchyma is lost because of infection, heart attack, or arteriosclerotic ischemia.^29–31^ Since elderly patients already have sinus lipomatosis (i.e., an accumulation of excess nontumorous fatty tissue in the renal sinus) as a natural process,^5^ it should be considered by readers that our oldest study patient was only 46 years old.

There remain many methods for the measurement of FRS.^3,29,32,33^ Numerical and volumetric measurements can be made. We preferred numerical measurements for this study.^34^ In this study of FRS differences, there was a statistically significant difference between the steatosis and non-steatosis subgroups. (p = 0.02) Based on our results and those from earlier studies,^35–37^ the width of FRS may be a useful auxiliary finding to assess early HS stages.

### Study limitations

Our smaller sample study had several limitations. Patients with HS were overwhelmingly males compared to the non-steatosis group. Due to our sample size, we didn’t stratify our sample patients by gender or body mass index (BMI).

## CONCLUSIONS

Based on these results, the measurement of liver and spleen HU and FRS widths during CT of younger adults can be a useful auxiliary method to assess lower grade HS stages. Future investigations of these relationships with larger heterogenous samples of patients undergoing radiological and pre or post-radiological primary care evaluation and the testing of more precise measurement criteria for possible FLD are certainly indicated.

### Conflict of interest

The authors have no conflict of interest to declare.

### Financial disclosure

The authors have no financial interest to declare.
